# Pulsed Electric Fields (PEF) and Accelerated Solvent Extraction (ASE) for Valorization of Red (*Aristeus antennatus*) and Camarote (*Melicertus kerathurus*) Shrimp Side Streams: Antioxidant and HPLC Evaluation of the Carotenoid Astaxanthin Recovery

**DOI:** 10.3390/antiox12020406

**Published:** 2023-02-07

**Authors:** Ana Cristina De Aguiar Saldanha Pinheiro, Francisco J. Martí-Quijal, Francisco J. Barba, Ana M. Benítez-González, Antonio J. Meléndez-Martínez, Juan Manuel Castagnini, Silvia Tappi, Pietro Rocculi

**Affiliations:** 1 Department of Agricultural and Food Science, Campus of Food Science, Alma Mater Studiorum, University of Bologna, 47521 Cesena, Italy; 2 Department of Preventive Medicine and Public Health, Food Science, Toxicology and Forensic Medicine, Faculty of Pharmacy, Universitat de València, Avda. Vicent Andrés Estellés, s/n, Burjassot, 46100 València, Spain; 3 Food Colour and Quality Laboratory, Facultad de Farmacia, Universidad de Sevilla, 41012 Sevilla, Spain; 4 Interdepartmental Centre for Agri-Food Industrial Research, Alma Mater Studiorum, University of Bologna, Via Quinto Bucci, 336, 47521 Cesena, Italy

**Keywords:** crustacean side streams, valorization, bioactive compounds, extraction, emerging technologies

## Abstract

Shrimp side streams represent an important natural source of astaxanthin. Optimization of the astaxanthin extraction process from shrimp side streams is of great importance for the valorization of crustacean side streams and the development of astaxanthin-related products. The combined and independent effects of two innovative extraction technologies (pulsed electric fields (PEFs) and accelerated solvent extraction (ASE)) alone and/or combined in a sequential step, using two different solvents on astaxanthin extraction from two shrimp species, were evaluated. Astaxanthin content in the extracts of shrimp side streams was determined by both spectrophotometric and HPLC assays, being the determination of the carotenoid profiles performed by HPLC analysis. Compared to a solvent extraction control procedure, the astaxanthin content was increased after ASE and PEF treatments, for both shrimp species, independently of the solvent used. The highest recovery (585.90 µg/g) was obtained for the species *A. antennatus*, with the solvent DMSO when PEF and ASE were combined, while the increase in antioxidant capacity varied depending on the solvent used. HPLC analysis of the samples revealed the presence of unesterified (all-E) astaxanthin, four unesterified Z isomers of astaxanthin and many unresolved astaxanthin esters. Both technologies are useful tools to recover antioxidant valuable carotenoids such as astaxanthin from shrimp side streams.

## 1. Introduction

Crustacean wastes are important side streams obtained from seafood processing. Each year, ~6–8 million tons of crustacean waste is generated worldwide [1]. Shrimp and crab are one of the most important internationally traded seafood products, being one of the few which can be considered as a “commodity”, valued at USD 10 billion (or 16% of global fisheries exports) [2].

Shrimp side streams currents provide an important natural source of antioxidant carotenoids, of which astaxanthin (ASX) is the most important one. ASX is one of the most important carotenoids in terms of market, largely to their use as a feed additive [3,4]. The carotenoid content of crustaceans can vary significantly, with significant differences due to the number of carotenoids found according to the feed, environmental conditions and animal species, as well as the extraction methods used [5].

Solvent extraction is the most popular method for ASX recovery, and while most of the studies focus on the screening of conventional solvents, the extraction yield remains low. There are several organic solvents commonly used to extract natural ASX from plant and animal tissues, including ethanol, chloroform, acetone, methanol and dimethyl sulfoxide (DMSO) [6]. The solvent DMSO has good permeability in many cell types, and many studies have already reported that it improves the recovery of carotenoids from microalgae and plants [7,8,9,10,11]. In addition, Wang and Meng et al. [8] reported that dimethyl sulfoxide (DMSO) used for the extraction of astaxanthin from *H. pluvialis* resulted in accurate spectrophotometric quantification of astaxanthin. Ethanol is a commonly used solvent for extraction that is also suitable for use in the food industry; however, the yield for the extraction of carotenoids from microalgae is low compared to DMSO [12]. Recently, different strategies to increase the carotenoid recovery efficiency in a sustainable way have been investigated, for example, oil-soluble methods, ultrasound, microwaves and enzyme-assisted extraction, the use of supercritical fluids and their combination [13,14,15,16]. The optimization of the extraction process of ASX from shrimp shells is of great importance for the valorization of crustacean side streams and the development of astaxanthin-related products [5].

Currently, pulsed electric fields (PEFs) technology is extensively employed in the food industry as an innovative processing technology [17], being a promising strategy for the tailored recovery of several components from seafood side streams, such as protein (i.e., collagen), minerals (i.e., calcium), chondroitin sulphate, chitosan, etc. [18,19]. On the other hand, Accelerated Solvent Extraction (ASE), also known as Pressurized Liquid Extraction (PLE), is considered as a green technique, which can be efficiently used to recover high-added-value compounds from food matrices and side streams [20]. Recently, some studies have investigated the application of ASE to obtain aqueous protein extracts with in vitro antioxidant capacity from fish (rainbow trout, sole, sea bass, sea bream and salmon) side streams [21,22,23]. Despite several publications dealing with the recovery of compounds from crustacean side streams, the application of both technologies for this purpose is still scarce [5], and to our knowledge, there are no publications combining PEF and ASE in a sequential process, specifically for this purpose. The main aim of this work is to apply PEF and ASE, alone and/or combined using dimethyl sulfoxide (DMSO) and ethanol as an extraction solvent, to recover ASX from shrimp side streams and to evaluate the effects of these technologies, alone or combined on the ASX content and antioxidant activities of the extracts obtained.

## 2. Materials and Methods

### 2.1. Sample Preparation

Red shrimp (*Aristeus antennatus*) and camarote prawn (*Melicertus kerathurus*) fresh samples were purchased from a local market (Valencia, Spain), during May 2021 and then transported refrigerated to the University of Valencia. Shrimps were processed by removing the head and body shell (carapace). The side streams (head and shells) were weighted and frozen at −40 °C for 48 h. Afterward, they were freeze-dried (2.5 FREEZONE, LABCONCO, Kansas City, MO, USA) for 72 h. Freeze-dried samples were ground and frozen at −25 °C until the extraction process. A portion of fresh shrimp side streams was refrigerated for some hours at 4 °C until PEF treatment, being frozen and freeze-dried after PEF treatment as described above for the other samples.

### 2.2. Chemicals and Reagents

Potassium persulfate (K_2_S_2_O_8_), AAPH (2,2′-azobis-2-methyl-propanimidamide), fluorescein sodium salt, Trolox (6-hydroxy-2,5,7,8-tetramethylchroman-2-carboxylic acid), ABTS (2,2′-azinobis (3-ethylbenzothiazoline 6-sulfonic acid), diatomaceous earth (Hyflo^®^Super Cel^®^), and astaxanthin standard were bought from Sigma-Aldrich (Steinheim, Baden-Württemberg, Germany). Dimethyl sulfoxide (DMSO) and absolute ethanol were provided by VWR International Eurolab S.L. (Barcelona, Spain). 

### 2.3. Process Description

Dimethyl sulfoxide (100% DMSO) and absolute ethanol were used as organic solvents, and four different extraction processes for each type of solvent were evaluated for ASX extraction from the side streams of each shrimp species:

1. ASE with DMSO and ASE with ethanol;
2. ASE using samples pretreated with PEF (PEF + ASE);
3. PEF-assisted extraction with DMSO and PEF-assisted extraction with ethanol;
4. Solvent extraction with DMSO (control) and solvent extraction with ethanol (control)/

### 2.4. Extraction Processes

#### 2.4.1. Solvent Extraction

After PEF treatment, ASX was extracted from shrimp side streams by solvent extraction. In this study, we chose ethanol and DMSO to compare the efficiency of these two different solvents for the extraction of ASX from shrimp side streams. Solvent extraction was performed according to the method proposed by Kokkali et al. [24]. In the solvent extraction, the samples were added to the solvent (1:10, *w*/*v*) and stirred at 400 rpm for 30 min at room temperature. Samples were then centrifuged at 4000 rpm for 10 min using a 5810R centrifuge (Eppendorf AG, Hamburg, Germany). The supernatant was collected and frozen at −25 °C. In parallel, a control sample (without PEF pretreatment) was also extracted using the same solvent extraction method and its yield was compared with that of the innovative extraction methods (ASE, PEF-assisted extraction and PEF + ASE). Both solvent extraction processes were performed in triplicate.

#### 2.4.2. Pulsed Electric Fields (PEFs) Treatment

PEF treatment conditions were previously decided in preliminary experiments (data not shown). The PEF machine used was PEF-Cell crack III (German Institute of Food Technologies (DIL) equipment (ELEA, Quakenbrück, Osnabrück, Germany)), located at the Faculty of Pharmacy of the University of Valencia (València, Spain). PEF treatment was carried out between two plate electrodes at a distance of 10 cm, and the corresponding electric field strength *E* was 3 kV/cm. The specific energy was 100 kJ/kg, and the number of pulses was 74. Fresh side streams (30 g) were placed into the processing chamber, and 300 mL of tap water was added. Before and after treatment, the temperature and conductivity in the sample were measured using a portable conductivity meter ProfiLine Cond 3310 (WTW, Xylem Analytics, Weilheim in Oberbayern, Germany). After PEF treatment, samples were frozen and freeze-dried as previously described. Samples pretreated with PEF were extracted by solvent extraction (PEF + Solvent) and ASE (PEF + ASE). 

#### 2.4.3. Accelerated Solvent Extraction (ASE) Process

Similarly, ASE optimal extraction conditions were also previously selected in preliminary experiments (Table S1). The accelerated solvent extractor ASE 200 Dionex (Sunnyvale, CA, USA) was used for the extraction of ASX from shrimp side streams. Thus, 1 MPa of nitrogen was used to purge the cells and assist the pneumatic system. DMSO and ethanol were the extracting solvents. The standard operating conditions were 1 min of preheating period, 5 min of heating period, 60% of flush volume, 10 MPa of extraction pressure (for 15 min) and 60 s of nitrogen purge. The temperature used was 50 °C. The dried samples were mixed with diatomaceous earth (DE) at a ratio of 1:2 (sample: DE, *w*/*w*)) before extraction. A glass fiber filter was placed in the end part of 22 mL pressure-resistant stainless-steel cells, in which the extractions were performed. The obtained extracts were distributed into several replicates and were kept at −25 °C for further analysis.

### 2.5. Determination of Astaxanthin Content

#### 2.5.1. Spectrophotometric Analysis

The determination of carotenoids in the extracts, reported as astaxanthin, was evaluated by measuring the absorbance of the appropriately diluted extracts at 470 nm using a spectrophotometer Perkin-Elmer UV/Vis Lambda 2 (Perkin-Elmer, Rodgau-Jügesheim, Germany). The content of carotenoids was calculated as astaxanthin [25] using Equation (1):

(1) $$C\;(µg/g\;\mathrm{samples})=\frac{A_{470\;\mathrm{nm}}\cdot \;V_{\mathrm{extract}}\;\cdot \;dilution\;factor}{\;W_{\mathrm{sample}}\;}$$

where *A*_470 nm_ is the maximum absorbance, *V*_extract_ is the volume of the extract and *W*_sample_ is the weight of the sample. A standard curve of astaxanthin was prepared following the method described by [26] with slight modifications. Each extraction was performed in duplicate and analyzed twice.

#### 2.5.2. HPLC Analysis

For this purpose, 2 mL of the samples (that is, the different extracts obtained) was taken, and 2 mL of dichloromethane and 10% sodium chloride were added to 8 mL, vortexed for 5 min and centrifuged (18,000× *g*) at 20 °C for 5 min. The lipophilic phase at the bottom was recovered. Two additional washes with sodium chloride were performed according to the same protocol, and the pooled extracts were concentrated to dryness in a rotary concentrator under vacuum (Eppendorf Concentrator Plus, Hamburg, Germany). The dry extracts were dissolved in ethyl acetate and an HPLC analysis was carried out using a previously validated method development by Stinco et al. [27] on an Agilent 1260 System (Agilent, Palo Alto, CA, USA) equipped with a DAD. The carotenoids were separated on a YMC C30 column (5 μm, 250 × 4.6 mm) (YMC, Wilmington, NC, USA), which was kept at 20 °C. A mobile phase consisting of methanol, methyl tert-butyl ether and water pumped at 1 mL/min was used with a linear gradient elution described elsewhere [27]. Astaxanthin was identified by comparing the chromatographic and UV/vis spectroscopic characteristics with those of a commercial standard (Sigma-Aldrich, Steinheim, Baden-Württemberg, Germany). This was stereomutated by heating based on a common methodology [28] to obtain a mixture of geometrical isomers of astaxanthin that allowed for the identification of the different isomers in the samples. The chromatographic and UV/Vis spectroscopic characteristics of the isomers are summarized in Table 1.

### 2.6. Antioxidant Capacity

The Trolox Equivalent Antioxidant Capacity (TEAC) assay was carried out according to the method previously described by Re et al. [29]. An ABTS+ radical stock solution containing 7.4 mM ABTS (2,2′-azino-bis (3-ethylbenzohiazoline-6-sulfonic acid) and 2.6 mM potassium persulfate dissolved in water was prepared and kept under darkness for 12–16 h before use. The working solution was prepared by mixing the ABTS+ stock solution with ethanol to obtain an absorbance of 0.70 ± 0.01 at 734 nm. The assay was performed by mixing 3 mL of the ABTS+ working solution with 30 µL of appropriately diluted extracts (1:2, *v*/*v*) and determining the absorbance after 6 min. The analyses were performed in triplicate and the antioxidant activity was calculated using a Trolox standard curve and expressed in µM Trolox equivalents. In addition, the Oxygen Radical Absorbance Capacity (ORAC) method was performed as previously described by Barba et al. [30]. An automated ORAC assay was performed using a VICTOR3 1420 multilabel plate counter (PerkinElmer, Turku, Finland) with fluorescence filters for an excitation wavelength of 485 nm and an emission wavelength of 535 nm. Sodium fluorescein and AAPH solutions were used at a final concentration of 0.015 and 120 mg/mL, respectively. Trolox (100 µM) was used as antioxidant standard, and samples were properly diluted with phosphate buffer (75 mM, pH 7). The assays were performed in a white 96-well flat-bottom plate. To each well, we added 50 µL of the diluted sample, Trolox standard or phosphate buffer (blank), 50 µL of fluorescein and 25 µL of AAPH. Fluorescence was recorded every 5 min for a period of 60 min (until fluorescence in the assay was less than 5% of the initial value). Each extract was analyzed in five replicates. Results were calculated considering the differences of areas under the fluorescence decay curve (AUC) between the blank and the sample over time and were expressed as µM Trolox Equivalents. 

### 2.7. Statistical Analysis

One-way analysis of variance (ANOVA) test was used to determine significant differences among samples. Moreover, a Tukey’s post test was applied with a significance level of *p* < 0.05.

## 3. Results and Discussion

### 3.1. Astaxanthin Content

**Figure 1:**
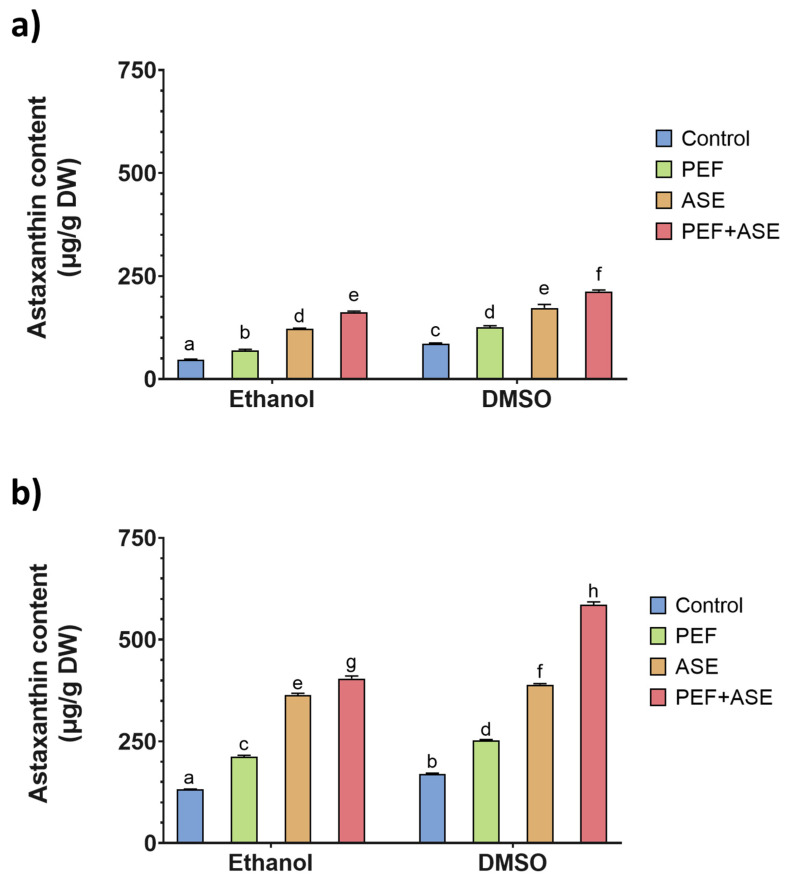
Astaxanthin content determined by HPLC for *M. kerathurus* (**a**) and *A. antennatus* (**b**) side streams. Pulsed electric fields (PEFs). ASE (Accelerated Solvent Extraction). Different letters above the bars indicate statistically significant differences between treatment averages (*p* < 0.05).

The ASX content in the extracts from shrimp side streams was determined by both spectrophotometric and HPLC assays. The results were correlated, observing no significant differences between the results obtained by both assays. In Figure 1a and Figure 1b are shown the astaxanthin values obtained by the HPLC assay for *M. Kerathurus* and *A. antennatus*, respectively. No significant differences were observed between the values of total ASX determined by both assays, ranging the values of shrimp side streams from 47.3 ± 1.2 μg/g on a dry weight basis (dw) to 213.1 ± 3.4 μg/g dw and from 169.6 ± 2.6 μg/g dw to 585.90 ± 6.9 μg/g for content of ASX for *M. kerathurus* and for *A. antennatus*, respectively. As was previously discussed by other authors, the ASX content in crustaceans can differ substantially, due to modifications in carotenoid amounts available in the feed, species, environmental conditions and body parts as well as extraction methods and solvent used [31,32]. In another study, Owaga et al. [33] found total carotenoid levels of 47.1 µg/g waste dw from heads of Brazilian shrimp (*Litopenaeus vannamei*). Moreover, Dave et al. [34] found values of astaxanthin of 284.48 μg/g in Atlantic shrimp (*Pandalus borealis*) side streams after using a mixture of hexane/isopropanol (3:2, v:v) for the extraction. In addition, Takeungwongtrakul et al. [35] observed the highest carotenoid content (378.95 mg/kg) recovery from the hepatopancreas of *Litopenaeus vannamei* by using isopropanol:hexane (50:50 (*v*/*v*)). 

ASX contents among the two shrimp species were significantly different (*p* < 0.05), regardless of the extraction procedure. *A. antennatus* had the highest astaxanthin content (from 169.6 ± 2.6 μg/g dw to 585.9 ± 6.9 µg/g dw), regardless of the extraction method used, while the maximum value for *M. kerathurus* was 213.1 ± 3.4 µg/g dw. However, considering the values from the literature, the content found in both species can be considered high, highlighting the potential of valorizing shrimp processing side streams into high-added-value ASX products. Moreover, the results obtained showed that both extraction pretreatment methods used had significant effects on the recovery of ASX content. Regardless of shrimp species, PEF and ASE technologies independently or in combination significantly improved ASX extraction compared to the control.

In particular, PEF treatment followed by solvent extraction increased recovered astaxanthin in *M. kerathurus* and *A. antennatus* by 46% and 48%, respectively, compared to the respective control. 

In both shrimp species, ASE allowed us to extract significantly higher carotenoid amounts than PEF in combination with solvent extraction (*p* < 0.05). The application of ASE resulted in an approximately 3-fold increase in the amount of astaxanthin extracted compared to the control, while in the shrimp species with the lowest carotenoid content (*M. kerathurus*), the application of ASE resulted in an approximately 2-fold increase in the amount of astaxanthin extracted compared to the control. Finally, the extraction with DMSO recovered a higher amount of astaxanthin than ethanolic extraction, independently of the extraction method used. In the food industry, various organic solvents, such as hexane, isopropanol, acetone, methanol and ethanol, have been widely used for the extraction of astaxanthin [6]. It has been previously reported that DMSO facilitates the extraction of carotenoids from microalgae [7,9]. Then, both techniques allowed us to increase the recovery of bioactive compounds such as astaxanthin compared to conventional extraction. Therefore, both solvent and time are put to better use, contributing to a more efficient use of resources and reducing the total volume of solvent required, which is related to sustainable development strategies [36].

In some previous studies, Gulzar and Benjakul [37] used PEF pretreatment in combination with an ultrasound-assisted process (UAE) to extract lipids and carotenoids from the cephalothorax of shrimp (*Litopenaeus vannamei)* (electric field strength: 4–16 kV/cm, 120–240 pulses). The results obtained by these authors showed that the treatments maximized the lipid yield (30.34 g/100 g) and caused an increase in the content of PUFAs and carotenoids. Recently, a response surface design was applied to study the effects of temperature (46–114 °C), pressure (43–77 bar) and extraction time (7–24 min) on the recovered ASX from shrimp side streams. The results showed that the maximum ASX yield of 24 mg/kg of shrimp side streams was obtained at 87 °C (extraction temperature), 49 bar (pressure) and 14 min of extraction time [38]. 

In the present study, ASE combined with PEF pretreatment showed to be the best process for extracting ASX from shrimp side streams, maximizing the carotenoid content of the extracts. This improvement can be attributed to the generation of pores in the matrix after PEF treatment, which facilitate deep solvent penetration and increase pigment extraction, especially when ASE was applied. In the ASE process, the strong interaction force between the solute and the matrix can be significantly reduced at high temperatures and high pressure. The use of these particular pressure and temperature conditions leads to a change in the physicochemical properties of the solvent, including mass transfer, while at the same time decreasing the surface tension and viscosity of the solvent and increasing the solubility of the analyte. This allows the solvent to penetrate more easily and deeply into the solid matrix to be extracted, which significantly increases the extraction yield compared to conventional extractions [39]. The highest carotenoid content observed in *M. kerathurus* (213.1 ± 3.4 µg/g dw) and *A. antennatus* (585.9 ± 6.9 µg/g dw) was achieved using PEF pretreatment combined with the ASE process and DMSO as the solvent.

**Figure 2:**
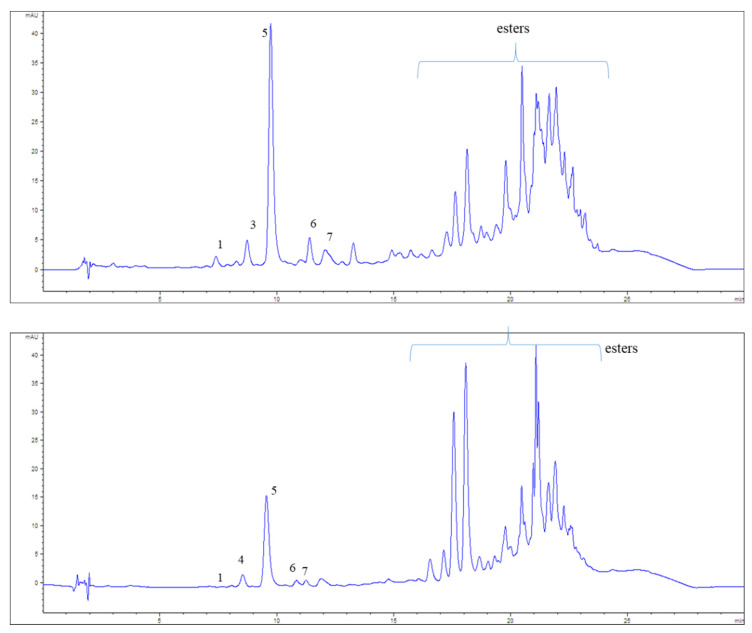
Profile chromatograms at 470 nm for Red (*Aristeus antennatus*) and Camarote shrimp (*Melicertus kerathurus*) extracts. Chromatographic and UV/Vis spectroscopic data can be found in Table 1.

Although there were no significant differences between the values measured by the two assays for total ASX content, the HPLC method provided relevant information because most studies assessed total ASX content using only the spectrophotometric assay. Moreover, there is a lack of information on the ASX geometrical isomers, which are really important because they may have different physicochemical properties, which, in turn, lead to differences in aspects, such as functionality or bioavailability [40]. In addition, some geometric isomers may also be markers of technological processes since carotenoids are predominantly found in their all-*trans* configuration [41]. However, extraction processes could lead to the formation of *Z* isomers that have different biological properties, which would alter their bioavailability and antioxidant capacity. In this line, the HPLC analysis of the samples was carried out, revealing the presence of unesterified (all-*E*)-astaxanthin, four unesterified *Z* isomers of astaxanthin and many unresolved astaxanthin esters (Figure 2).

The conclusive identification of Z isomers of carotenoids is difficult in most cases due to the lack of commercially available standards and the need to use techniques such as NMR. The tentative identification of geometrical isomers of ASX is especially challenging as its UV/Vis spectra lack a fine structure. Therefore, the common approach is the identification based on spectroscopic features (e.g., hypsochromic shift relative to the all-*E* isomer, the height of the *cis* peak, the loss of fine structure) [28] Considering the hypsochromic shift (to shorter wavelengths) of the absorption maxima of *Z* isomers relative to the all-*E* isomer (which is ~2–6 nm for mono-Z isomer and higher for di-*Z* or poli-*Z* isomers) [42], it may be hypothesized that peaks 3, 5 and 6 correspond to mono-*Z* isomers of astaxanthin and peak 4 to a di-*Z* isomer. In any case, the free all-*E* isomer predominated over the free *Z* isomers.

**Figure 3:**
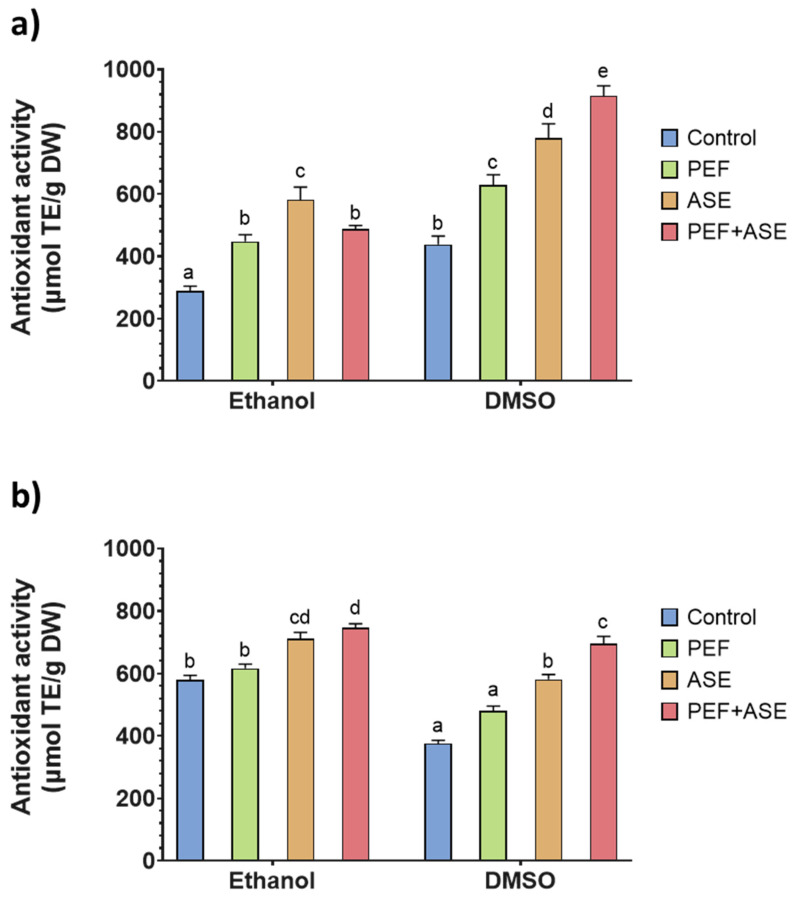
Trolox equivalent antioxidant capacity (TEAC) of the extracts from *M. kerathurus* (**a**) and *A. antennatus* (**b**) side streams. Different letters above the bars indicate statistically significant differences between treatment averages (*p* < 0.05).

On the other hand, xanthophylls (oxygenated carotenoids) such as astaxanthin can be free or associated in different ways, including the esterification with fatty acids, which can markedly properties, such as solubility or susceptibility to oxidation, among others. This, in turn, can result in changes in aspects, such as stability or bioavailability [43]. Being more lipophilic, carotenoid esters elute later than the carotenoid-free forms. As can be inferred from Figure 3, where many late-eluting unresolved peaks were observed, astaxanthin was predominantly esterified in the samples. The esterification of astaxanthin has been studied in the microalgae *Haematococcus pluvialis*, krill and shrimp [44,45].

### 3.2. Antioxidant Capacity

**Figure 4:**
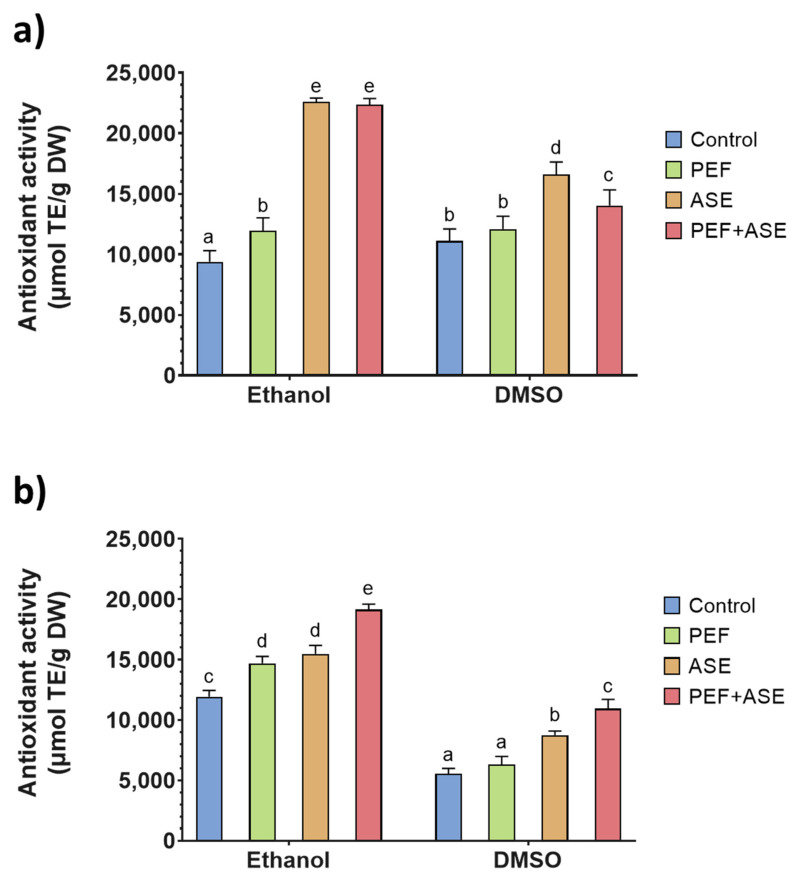
Oxygen Radical Absorbance Capacity Assay (ORAC) of the extracts from *M. kerathurus* (**a**) and *A. antennatus* (**b**) side streams. Different letters above the bars indicate statistically significant differences between treatment averages (*p* < 0.05).

Trolox equivalent antioxidant capacity assay (TEAC) and oxygen radical absorbance capacity (ORAC) of shrimp side stream extracts were used to evaluate the antioxidant capacity of the extracts rich in astaxanthin, and the results are shown in Figure 3 and Figure 4, respectively.

All extracts from shrimp side streams exhibited notable ABTS+ radical scavenging activity in TEAC assay. However, significant differences (*p* < 0.05) were observed among results obtained using different extraction processes and solvent for each species. 

For *M. kerathurus*, the TEAC values were in a range of 437.9 ± 27.1 to 915.4 ± 31.8 µmol TE/g dw (Figure 3a), while for *A. antennatus*, TEAC values ranged from 375.8 ± 12.4 up to 693.7 ± 25.5 µmol TE/g dw (Figure 3b). In both species, the maximum values were found for samples pretreated with PEF and extracted by ASE (PEF + ASE) but, for *A. antennatus*, higher TEAC values were achieved when ethanol was used, while the best results for *M. kerathurus* were obtained with DMSO extraction. Considering the individual treatments, both ASE and PEF processes significantly increased the TEAC values (*p* < 0.05) for *M. kerathurus*, while for *A. antennatus*, PEF processes had no significant effect (*p* > 0.05) compared with control. 

On the other hand, the ORAC assay is one of the most common methods for assessing peroxyl radical ROO▪ scavenging capacity [46]. The ORAC values varied from 5567 ± 424 µmol TE/g dw to 22,600 ± 306 µmol TE/g dw for *A. antennatus* (Figure 4). 

The highest ORAC values were observed in the extracts of *M. keranthurus* extracted by ASE (22,600 ± 306 µmol TE/g) and of *A. antennatus* extracted by ASE + PEF (10,944 ± 768 µmol TE/g) using, in both cases, ethanol as the solvent. The higher ORAC value of the *A. antennatus* extracts found in samples extracted by ASE + PEF was in accordance with the highest ABTS+ scavenging activity. 

In general, it was observed that *M. kerathurus* extracts showed a higher ORAC value than *A. antennatus* extracts, although this species showed significantly lower (*p* < 0.05) astaxanthin values than *A. antennatus*. Shrimp side streams contain astaxanthin and its esters as the major pigments [47], and their antioxidant activity is well documented. However, shrimp by-product extract contains other antioxidants, such as phenolics, in addition to carotenoids [48]. Moreover, crustaceans are rich in several other lipophilic antioxidants, such as tocopherol and ubiquinol [49]. The simultaneous presence of other antioxidants also affects the antioxidant potential of the extracts, since antioxidants are known to have a synergistic effect [50,51]. As shown by the results of the present study, the ethanolic extracts have higher antioxidant capacity, as measured by TEAC and ORAC, especially for the extracts of *A. antennatus*. However, the yield of ASX extraction was higher when DMSO was used as the solvent. For this reason, it can be hypothesized that, on the one hand, ethanol was able to extract antioxidant compounds other than ASX and at a higher concentration than DMSO. In addition, components other than carotenoids may have affected the free radical scavenging activity of the extracts. Moreover, TEAC and ORAC are based on different antioxidant activity mechanisms. Despite both assays using Trolox as a reference antioxidant and expressing results based on Trolox equivalents, results obtained for TEAC and ORAC may lead to different conclusions, in agreement with the data reported in the literature [52]. Therefore, to better understand the results obtained, a more comprehensive characterization of the extract should be performed.

## 4. Conclusions

For the first time, the effect of applying the innovative technologies ASE and PEF alone or in combination to recover ASX from shrimp side streams was investigated. This study shows that the application of the proposed technologies increased the ASX content in the extracts for both shrimp species and their effect was maximized when they were used in combination. Both techniques are environmentally friendly and safe and appear to be an effective means to obtain astaxanthin-rich extracts with strong antioxidant activity from shrimp side streams. However, these techniques are still poorly developed and adapted to the application of shrimp side streams due to a lack of standardization on an industrial scale. These promising results should be confirmed by extending the investigation to other valuable compounds from crustacean side streams.

## Figures and Tables

**Table 1:** Chromatographic and UV/Vis spectroscopic characteristics of geometrical isomers of astaxanthin.

Number	Name	Retention Time (Minutes)	Maximum Wavelength (nm)
1	Z-isomer	7.50	465
2	Z-isomer	7.51	458
3	Z-isomer	7.91	368
4	Z- isomer	9.18	370
5	(all-*E*)-astaxanthin	10.18	477
6	Z-isomer	11.06	470
7	Z-isomer	13.07	470

## Data Availability

Data will be available under request.

## Supplementary Materials

The following supporting information can be downloaded at: https://www.mdpi.com/article/10.3390/antiox12020406/s1, Table S1: Astaxanthin recovery from shrimp side streams after accelerated solvent extraction (ASE) using different temperatures and solvents.
