# Supplementary material for: Pulsed Electric Fields (PEF) and Accelerated Solvent Extraction (ASE) for Valorization of Red (Aristeus antennatus) and Camarote (Melicertus kerathurus) Shrimp Side Streams: Antioxidant and HPLC Evaluation of the Carotenoid Astaxanthin Recovery
Source: Antioxidants (Basel). 2023 Feb 7;12(2):406. doi: 10.3390/antiox12020406 (PMC9951945; doi:10.3390/antiox12020406)
Supplement: Supplementary file 1 [file antioxidants-12-00406-s001.zip › antioxidants-2171933-supplementary corrected.pdf]

Table S1. Astaxanthin recovery from shrimp side streams after accelerated solvent extraction (ASE) using different temperatures and solvents.

Shrimp Specie	Solvent	Temp. (°C)	Pressure (bar)	Time (min)	ASX (µg/g dw)	TEAC (µmol TE/g dw)
M. kerathurus	Ethanol	40	103.42	15	94.81 ± 1.73^{a}	543.33 ± 14.88^{ab}
		50			121.98 ± 1.53^{b}	582.11 ± 22.40^{b}
		60			126.09 ± 4.78^{b}	521.37 ± 17.12^{a}
	DMSO	40			139.56± 1.61^{a}	614.61± 33.35^{a}
		50			172.99± 8.42^{b}	779.99± 36.23^{c}
		60			168.44± 2.55^{b}	697.93± 16.33^{b}
A. antennatus	Ethanol	40			239.40 ± 4.09^{a}	651.40 ± 35.25^{b}
		50			364.22 ± 6.38^{b}	711.52 ± 19.41^{c}
		60			365.14 ± 7.96^{b}	664.52 ± 25.37^{a}
	DMSO	40			286.65 ± 3.06^{a}	608.74 ± 18.45^{b}
		50			389.73 ± 2.43^{c}	581.20 ± 13.73^{b}
		60			357.70 ± 11.47^{b}	489.98 ± 13.19^{a}

Different letters in superscript by column indicate statistically significant differences in the concentration of astaxanthin (ASX) and antioxidant capacity (TEAC) between extraction conditions for each type of shrimp ($p < 0.05$).
